# Weight‐related lifestyle behaviours and the COVID‐19 crisis: An online survey study of UK adults during social lockdown

**DOI:** 10.1002/osp4.442

**Published:** 2020-08-12

**Authors:** Eric Robinson, Steven Gillespie, Andrew Jones

**Affiliations:** ^1^ Department of Psychological Sciences University of Liverpool Liverpool UK

**Keywords:** COVID‐19, lifestyle behaviours, obesity, weight management

## Abstract

**Background:**

The COVID‐19 crisis is likely to have had wide‐ranging consequences on lifestyle behaviours and may have affected weight management. The objective of the present study was to examine perceptions of how weight‐related lifestyle changed in social lockdown among UK adults compared with before the emergence of the COVID‐19 crisis.

**Methods:**

As part of an online cross‐sectional survey conducted during social lockdown in the United Kingdom, 723 UK adults reported on the extent to which their eating (healthiness of diet, frequency of bingeing on food), physical activity, sleep and alcohol consumption had changed since the emergence of the COVID‐19 crisis and completed measures of current psychological well‐being.

**Results:**

Although both improvements and declines in weight gain protective behaviours were reported, 79% of participants reported a decline in one or more weight gain protective behaviours. Both participants with a diagnosis of psychiatric illness or obesity (body mass index [BMI] ≥ 30) were most likely to report declines in weight gain protective behaviours and show an overall profile of weight management behaviours worsening. Participants experiencing high levels of stress also reported reductions in more weight gain protective behaviours.

**Conclusions:**

Lifestyle behaviours associated with weight gain are likely to have been affected by the COVID‐19 crisis. Reductions to the perceived frequency by which people engage in behaviours usually associated with successful weight management appear to be common, and people living with obesity and mental health problems may be at increased risk.

## 1 INTRODUCTION

The emergence of the worldwide COVID‐19 crisis produced an immediate and substantial public health burden.^1^ During the early phases of the crisis, governments took measures to reduce the spread of COVID‐19, and many countries were placed under government‐enforced ‘lockdowns’. Such lockdown restrictions are likely to have wide‐ranging consequences for public health, as they invariably change movement, work, social and leisure time activities.^2^, ^3^, ^4^ For example, behaviours that are health protective and would normally be predicted to protect against weight gain, such as eating a heathy diet,^5^ not bingeing/overeating,^6^ remaining physically active,^7^ getting sufficient sleep^8^ and limiting alcohol consumption,^9^ may be more difficult to achieve during the COVID‐19 crisis. The current importance of weight‐related lifestyle behaviours is highlighted by research identifying higher body mass index (BMI) as a major risk factor for COVID‐19 hospitalization.^10^

The present research made use of data from UK adults collected during COVID‐19 lockdown in the United Kingdom. As part of the survey, participants retrospectively reported on the extent to which weight‐related lifestyle behaviours had changed or remained the same compared with before the COVID‐19 crisis. Patterns of perceived change across behaviours and whether specific population subgroups were more likely to report declines in lifestyle behaviours that would typically be weight gain protective were examined.

## 2 METHODS

Data were drawn from an online cross‐sectional survey that was conducted during 19–22 April 2020 (UK lockdown commenced 23 March). Participants were recruited from online panel provider Prolific Researcher,^11^ and the study was open to all panel members. Eligibility criteria were as follows: aged 18–60, UK resident and fluent in English. The survey contained a range of measures examining life during the COVID‐19 crisis, psychological well‐being, sexual behaviour, health behaviour, cognitive function and social circumstances. Further information is available in Supporting Information, and all questionnaire items collected as part of the wider survey are available at https://osf.io/jqmyd/. Two attention checks were included in the survey (e.g., ‘have you ever been to the planet Mars?’) in order to identify any participants not completing questionnaire items as intended. The study was approved by the University of Liverpool Research Ethics Committee and took approximately 15 min to complete.

### 2.1 Participant characteristics

Participants reported their age, gender, ethnicity, highest education level, household income and living circumstances, whether they had any high‐risk conditions for COVID‐19 (see Table 1), whether they had been diagnosed or suspected they had COVID‐19, and whether they had ever been diagnosed with a psychiatric illness (e.g., depression) and reported their weight and height. In line with the approach used in Cheng et al.,^12^ biologically implausible height (<111.8 or >228.6 cm) and weight values (<24.9 or >453.6 kg) and BMI values that were <12 or >70 kg/m^2^ were excluded.

**TABLE 1:** Sample characteristics

	*M* (*SD*) or %, *N* = 723
Gender (female)	488 (67%)
Age (years)	30.7 (9.6)
Ethnicity (White vs. not)	580 (80%)
Education (degree level or higher vs. not)	465 (64%)
Household income (mean £ per annum after tax)	£37,482 (£24,267)
High risk condition (≥1 vs. 0)	137 (19%)
Living alone (living alone vs. not)	73 (10%)
Psychiatric condition (previous diagnosis vs. not)	227 (31%)
COVID diagnosis (formal/suspected vs. not)	110 (15%)
Underweight (BMI, <18.5)	26 (4%)
Normal weight (BMI, 18.5–24.9)	370 (51%)
Overweight (BMI, 18.5–24.9)	179 (25%)
Obesity (BMI, ≥30)	148 (20%)
Loneliness (mean, *SD*)	22.8 (13.7)
Depression (mean, *SD*)	6.5 (5.3)
Anxiety (mean, *SD*)	3.1 (3.4)
Stress (mean, *SD*)	6.3 (4.7)

*Note*: Income data indicative of very large household incomes (>£100,000, equivalent of more than 3 times the national average) was recoded to £100,000. High risk conditions: pregnant; lung condition, such as asthma, COPD, emphysema or bronchitis, and heart disease, such as heart failure; chronic kidney disease; a liver disease, such as hepatitis; a condition affecting the brain and nerves, such as Parkinson's disease, motor neurone disease, multiple sclerosis (MS), a learning disability or cerebral palsy; diabetes; problems with spleen, for example, sickle cell disease, or if you have had your spleen removed; a weakened immune system as the result of conditions such as HIV and AIDS or medicines such as steroid tablets or chemotherapy; very overweight (having a BMI of 40 or above). COVID diagnosis: ‘yes’ responses to ‘Have you been formally diagnosed with COVID‐19? or ‘Do you suspect you have had or currently have COVID‐19? Loneliness is scored on a 0–60 scale. Depression, anxiety and stress are scored on a 0–21 scale, all with higher values indicating more.

Abbreviation: BMI, body mass index.

### 2.2 Lifestyle behaviours

Data were used from five items measuring weight‐related lifestyle behaviours; *Compared with before the COVID‐19 virus crisis, I have exercised/slept/eaten healthily/binged on food/drank alcohol’* and for each item participants responded on the same 1–7 scale (1 = a lot less than usual, 2 = less than usual, 3 = a little less than usual, 4 = about the same, 5 = a little more than usual, 6 = more than usual and 7 = a lot more than usual). For individual behaviours, a score of 1–3 was coded as a reduction in the behaviour, 4 = no change, and 5–7 coded as an increase.

### 2.3 Psychological measures

#### 2.3.1 Loneliness

Participants completed the 20‐item UCLA loneliness scale.^13^ Items measured feelings of loneliness (e.g., ‘I lack companionship’) during the last 2 weeks (*ω* = .95).

#### 2.3.2 Depression, anxiety and stress

Participants completed the DASS21,^14^ which included seven items on depression (*ω* = .91), anxiety (*ω* = .80) and stress (*ω* = .89) during the last week.

## 3 RESULTS

Nine hundred and seven participants were recruited into the study. One hundred and eighteen participants failed ≥1 attention check or provided duplicate responses, two provided implausible BMI data, and 64 were excluded due to missing data for variables used in the present analyses. The final analytic sample was *N* = 723. See Table 1 for sample characteristics.

Both reductions and increases in weight gain protective behaviours were common (see Table 2). For example, although 32% reported eating healthily less frequently, 30% reported eating healthily more frequently compared with before the COVID‐19 crisis. However, 49% of participants reported that they were bingeing on food more frequently, whereas only 19% reported this occurring less frequently. For both exercise (47%) and sleep (49%), it was most common for participants to report increases, although many reported exercising less (35%) and sleeping less (23%). The most common response for alcohol use was ‘remained the same’ (41%), and similar numbers reported increases (28%) and decreases (30%).

**TABLE 2:** Frequency of weight‐related lifestyle behaviours compared with before the COVID‐19 crisis

	No change	Less than before	More than before
Eating healthily	272 (38%)	231 (32%)	220 (30%)
Bingeing on food	235 (33%)	136 (19%)	352 (49%)
Exercising	133 (18%)	253 (35%)	337 (47%)
Sleep	200 (28%)	168 (23%)	355 (49%)
Alcohol consumption	299 (41%)	220 (30%)	204 (28%)

*Note*: Data from items compared with before the COVID‐19 virus crisis ‘I have exercised/slept/eaten healthily/binged on food/drank alcohol’. Response options: *a lot less than usual, less than usual, a little less than usual =* ‘less than before’. Response option*: about the same =* ‘no change’. Response options*: a little more than usual, more than usual, a lot more than usual =* ‘more than before’.

It was next examined how common it was for participants to report declines across the five protective behaviours (i.e., decreased: sleep, healthy eating and physical activity [vs. increased/remained same]; and increased: bingeing and alcohol use [vs. decreased/remained same]); 79% reported a decline in at least one protective behaviour. Frequencies were as follows: decline in 0 behaviours (*n* = 152, 21%), 1 (*n* = 213, 30%), 2 (*n* = 168, 23%), 3 (*n* = 117, 16%), 4 (*n* = 57, 8%) and 5 (*n* = 16, 2%). Linear regression analysis was conducted to predict variability in the number of protective behaviours that declined (0–5). The first step of the model included participant demographics. See Table 3. The second step included loneliness, depression, anxiety and stress. In Step 1, having a diagnosed psychiatric illness, higher BMI (both overweight and obesity BMI groups vs. normal weight) and higher household income were associated with declines in a greater number of behaviours. In Step 2, stress was associated with declines in a greater number of behaviours, and psychiatric illness diagnosis was no longer significant. Analyses were reran with participants with diagnosed/suspected COVID‐19 removed and using a Poisson regression, results were unchanged. For descriptive purposes, analyses for each of the individual behaviours and number of protective behaviours improved are reported in Tables S1–S5. Finally, because reports of both decreases and increases in weight gain protective behaviours were common, unplanned exploratory analyses on overall profile of change were conducted. Scores were totalled across all behaviours (reverse scoring: eaten healthily, exercised and slept) to create an index of overall change, with higher scores indicative of worsening of weight gain protective behaviours. Linear regression was used to examine whether participant characteristics predicted variability in the measure. As in the main analyses, having obesity or a previous psychiatric condition diagnosis was associated with less favourable overall change in behaviour. See Table 4. All analyses were conducted in SPSS24, and the data are available at https://osf.io/jqmyd/.

**TABLE 3:** Participant characteristics associated with number of weight gain protective behaviours reduced in frequency

	Step 1	Step 2
Gender	−.054 (±.11: 95% CI [−.26, .16]), *p* = .62	.010 (±.11: 95% CI [−.20, .22]), *p* = .93
Age	−.010 (±.01: 95% CI [−.02, < .00]), *p* = .06	−.004 (±.01: 95% CI [−.02, .01]), *p* = .45
Ethnicity	.163 (±.13: 95% CI [−.08–.41]), *p* = .19	.081 (±.12: 95% CI [−.16, .32]), *p* = .51
Education	−.033 (±.11: 95% CI [−.24, .17]), *p* = .75	−.039 (±.10: 95% CI [−.24, .16]), *p* = .70
Household income	.005 (±.00: 95% CI [<.00, .01]), *p* = .02^*^	.005 (±<.00: 95% CI [<.00, .01]), *p* = .01^*^
High risk condition	−.060 (±.13: 95% CI [−.32, .19]), *p* = .64	*−.*001 (±.13: 95% CI [−.25, .24]), *p* = .99
Living alone	.228 (±.17: 95% CI [−.09, .55]), *p* = .16	.283 (±.16: 95% CI [−.04, .60]), *p* = .08
Psychiatric condition	.238 (±.11: 95% CI [.03, .45]), *p* = .03^*^	.028 (±.11: 95% CI [.19, .25]), *p* = .80
COVID diagnosis	.154 (±.14: 95% CI [−.11, .42]), *p* = .26	.061 (±.14: 95% CI [−.21, .33]), *p* = .65
Underweight (BMI, <18.5)	.010 (±.27: 95% CI [−.51, .53]), *p* = .97	−.002 (±.26: 95% CI [−.51, .51]), *p* = .99
Overweight (BMI, 18.5–24.9)	.264 (±.12: 95% CI [.03, .50]), *p* = .03^*^	.245 (±.12: 95% CI [.01, .48]), *p* = .04^*^
Obesity (BMI, ≥30)	.349 (±.13: 95% CI [.09, .61]), *p* = .009^**^	.282 (±.13: 95% CI [.03, .54]), *p* = .03^*^
Loneliness	—	.002 (±.01: 95% CI [−.01, .01]), *p* = .70
Depression	*—*	.026 (±.02: 95% CI [−.01, .06]), *p* = .10
Anxiety	—	−.025 (±.02: 95% CI [−.07, .02), *p* = .24
Stress	—	.059 (±.02: 95% CI [.03, .09]), *p* = .001^**^
Model fit	*F*(12,710) = 2.21, *p* = .010	*F*(16, 707) = 4.75, *p* < .001
	Adj. *R* ^2^ = .020	Adj. *R* ^2^ = .077
Multicollinearity	*VIFs* > 1.03 < 1.26	*VIFs* > 1.08 < 3.27

*Note*: Values are unstandardized coefficients (± standard errors). Gender reference category = female (vs. male), ethnicity reference category = not White (vs. White), education is highest level of qualification with reference category = less than degree level (vs. degree level or higher), household income is in £1000/year after tax, high risk condition reference category = no condition (vs. one or more high risk conditions), living alone reference category = not alone (vs. alone), psychiatric condition reference category = no condition (vs. previous diagnosis), COVID diagnosis reference category = no diagnosis (vs. formally diagnosed or suspected). For loneliness, depression, anxiety and stress higher scores indicate increased levels. There were no influential cases in the regression as defined by Cook's distance >1.0.

Abbreviation: BMI, body mass index.

^*^ indicate significant at *p* < .05.

^**^ indicates significant at *p* < .01.

**TABLE 4:** Participant characteristics associated with overall change profile in weight gain protective behaviours (higher scores indicate worsening)

	Association with total score
Gender	*β* = .016, *p* = .687
Age	*β* = .052, *p* = .190
Ethnicity	*β* = .061, *p* = .112
Education	*β* = .002, *p* = .955
Household income	*β* = .048, *p* = .218
High risk condition	*β* = −.010, *p* = .804
Living alone	*β* = .068, *p* = .075
Psychiatric condition	*β* = .095, *p* = .014*
COVID diagnosis	*β* = −.007, *p* = .859
Underweight (BMI < 18.5)	*β* = − .011, *p* = .770
Overweight (BMI 18.5–24.9)	*β* = .037, *p* = .361
Obesity (BMI ≥ 30)	*β* = .084, *p* = .042^*^
Model fit	*F*(12,710) = 2.068, *p* = .017 Adj. *R* ^2^ = .017
Multicollinearity	*VIFs* >1.03 < 1.27

*Note*: Total score is sum across all five behaviours (reverse scoring: eaten healthily, exercised and slept) to create an index of overall change, with higher scores indicative of worsening of weight gain protective behaviours. Values are standardized coefficients. Gender reference category = female (vs. male), ethnicity reference category = not White (vs. White), education is highest level of qualification with reference category = less than degree level (vs. degree level or higher), household income is in £1000/year after tax, high risk condition reference category = no condition (vs. one or more high risk conditions), living alone reference category = not alone (vs. alone), psychiatric condition reference category = no condition (vs. previous diagnosis), COVID diagnosis reference category = no diagnosis (vs. formally diagnosed or suspected).

Abbreviation: BMI, body mass index.

^*^ Significant at *p* < .05.

## 4 DISCUSSION

In a sample of UK adults surveyed during social lockdown (April 2020), substantial numbers of participants reported changes to weight‐related lifestyle behaviours compared with before the emergence of the COVID‐19 crisis. Although declines in behaviours that are normally weight gain protective were common, some participants reported increases in weight gain protective behaviours (e.g., 47% reported exercising more frequently). The majority of the sample (79%) reported declines in frequency of at least one of the five weight gain protective lifestyle behaviours studied. A further aim was to examine whether participant characteristics were associated with declines in protective behaviours and adults living with overweight and obesity and a diagnosed psychiatric illness were significantly more likely to report declines. Living in a higher income household was also associated with perceived declines, although this effect was small in relative magnitude. Because reports of decreases and increases in weight gain protective behaviours were common, additional analyses examining overall profile of change across the five studied behaviours were conducted. Having obesity and a previous psychiatric diagnosis were both associated with a less favourable overall change in weight gain protective behaviour, suggesting that these groups may be at increased risk of weight gain during the COVID‐19 crisis.

The COVID‐19 crisis is likely to have a considerable burden on mental health.^15^ In the present study, higher stress levels during social lockdown were predictive of declines in weight gain‐related lifestyle behaviours, and after accounting for stress, participants with a diagnosis of a mental health illness were no longer at elevated risk of declines in behaviour. These findings highlight the need for psychological support to reach those at risk during the COVID‐19 crisis. Weight control efforts are normally more common in people with higher BMI,^16^ and a variety of changes caused by the COVID‐19 crisis (e.g., reduced access to healthy food) may be making weight control efforts more difficult among people with overweight/obesity. Because obesity is a risk factor for COVID‐19 hospitalization^10^ and risk of COVID‐19 infection is substantial, encouraging weight gain protective behaviours remains important. It is important to note that improvements in weight gain protective behaviours were also reported by many participants (e.g., 49% reported exercising more frequently), which may be due to changes in working patterns (e.g., removal of commuting time) and because exercise was one of the few permitted reasons to leave the home in the UK when data was collected.

In line with the online panel we recruited from,^11^ the sample was not representative of the wider UK population and was predominantly White. Both average household income and education level were somewhat higher, and the number of males and people with overweight and obesity were lower than in the United Kingdom,^17^ so results regarding income, ethnicity and education in particular should be interpreted cautiously. For these reasons, it will be important for further research to study how behaviours are changing in lower socio‐economic status groups and ethnic minorities. Likewise, those most affected by COVID‐19 (i.e., experiencing stress and financial difficulty) may be less likely to have participated. A limited number of lifestyle behaviours were studied and during a short time frame. More detailed studies of a wider range of behaviours (e.g., time spent sitting) over a longer period of time are needed. Participants reported on their perceptions of how their behaviour had changed. Measures therefore rely on retrospective recall, are subjective (e.g., definitions of ‘healthy eating’ will differ from person to person) and will be prone to bias. As an illustrative example, a person may have perceived that their levels of physical activity increased due to engagement with a new weekly exercise class, but in reality, their overall level of activity may have decreased due to COVID‐19 (e.g., reduction in active commuting or increase in sedentary time). Self‐report biases may also vary based on participant characteristics.^18^ For example, a person experiencing high levels of stress may be more likely to perceive negative changes in lifestyle behaviours. BMI was also based on self‐reported data. Notwithstanding these limitations, the present preliminary findings highlight the need to monitor weight change during the COVID‐19 crisis.

## CONFLICT OF INTEREST

ER has previously been the recipient of research funding from Unilever and the American Beverage Association for unrelated research.

## FUNDING

University of Liverpool funding provided to SG. ER is supported by the European Research Council.

## Supporting information


**Table S1:** Multinomial Logistic Regression predicting reported decline or increase in exercise compared to no change.
Table S2: Multinomial Logistic Regression predicting reported decline or increase in sleep compared to no change.
Table S3: Multinomial Logistic Regression predicting reported decline or increase in healthy eating compared to no change.
Table S4: Multinomial Logistic Regression predicting reported decline or increase in binge eating compared to no change.
Table S5: Multinomial Logistic Regression predicting reported decline or increase in binge eating compared to no change.

## Data Availability

The data analysed are openly available to researchers and accessible from the Open Science Framework.
