# Supplementary material for: Weight‐related lifestyle behaviours and the COVID‐19 crisis: An online survey study of UK adults during social lockdown
Source: Obes Sci Pract. 2020 Aug 12;6(6):735–40. doi: 10.1002/osp4.442 (PMC7746963; doi:10.1002/osp4.442)
Supplement: Supplementary file 1 — Table S1: Multinomial Logistic Regression predicting reported decline or increase in exercise compared to no change. Table S2: Multinomial Logistic Regression predicting reported decline or increase in sleep compared to no change. Table S3: Multinomial Logistic Regression predicting reported decline or increase in healthy eating compared to no change. Table S4: Multinomial Logistic Regression predicting reported decline or increase in binge eating compared to no change. Table S5: Multinomial Logistic Regression predicting reported decline or increase in binge eating compared to no change. [file OSP4-6-735-s001.docx]

Online Supplementary Materials

**Other measures in online survey**

For descriptive purposes below we provide detail on all others measures collected in the survey. See https://osf.io/jqmyd/ for the full survey administered to participants.

*Social Distancing*. Participants were asked in the previous two weeks how much time they had spent with friends, immediate family, colleagues and usual social network in person, with the anchors 1 (not at all) to 5 (very often). They were also asked about their social media use to connect / play games with friends and family, individuals/groups outside of their usual contacts using the same anchors.

*Coping Using Sex Inventory*. Participants completed the Coping Using Sex Inventory, which provides a series of scenarios (e.g. ‘I have fantasized about having sex with a consenting adult’) and Likert responses of 1 (not at all) to 5 (very often). Two versions of each scenario were asked; 1. In the two weeks before lockdown was introduced by the UK government (period from 10/03/2020 to 24/03/2020), and 2. In the previous two weeks.

*Difficulties in Emotional Regulation Short Form*. Participants complete the Difficulties in Emotional Regulation Short Form; a 16 item Likert scale focusing on behaviour over the previous two weeks (e.g. ‘When I have been upset in the past two weeks, I have become out of control.’), with anchors ranging from 1 (almost never) to 5 (almost always).

COVID19 Questionnaire Items. Participants were asked if they had been tested for and diagnosed with COVID19, and if they were currently symptomatic. They were also asked if they had not been for and diagnosed with COVID19 but thought they had had it and were symptomatic. Compared to before the pandemic they were asked how worried about their i) overall health, ii) financial security and iii) food access (a lot more worried, more worried, no more or no less worried, less worried, a lot less worried. Participants were asked about how their feelings and behaviours had changed since the pandemic (I have … felt lonely / felt depressed / felt anxious / intentionally harmed myself / had suicidal thoughts / exercised / slept / eaten healthily / binged on food / drank alcohol / smoked / experienced conflict with others / been verbally or physically abused by others / had poor physical health) on a 1 – 7 likert scale (1 = A lot less than usual, 7 = A lot more than usual).

*Go/No-Go task*. Participants complete a Go/No-Go task to measure inhibitory control. Each trial began with a fixation cross (‘+’) presented in the centre of the screen for 50 ms followed by a blank screen for 150 ms. Following this a shape (circle or square) appeared in a random spatial location on the screen for 1000 ms or until a response was made. On ‘Go trials’ participants were required to press space in response to the shape as quickly as possible, on ‘No-Go’ trials participants were required to withhold their response to the shape. Feedback was provided for omission errors (‘You should have pressed!’), commission errors (‘You should not have pressed!’), or if their reaction times on go trials was > 600 ms (‘Try to be faster!’). There were 5 experimental blocks each containing 50 trials (40 Go + 10 No-Go) and 1 practice block of 10 trials (8 Go + 2 No Go: not analysed). The mapping of Go and No-Go stimuli were presented at the beginning of each block. In the first three experimental blocks a circle was the Go response (square = No-Go). On the fourth block the rules changed, and the square signalled a Go response (circle = No-Go), which remained for the final block.

**Increases in protective behaviors**

For completeness we re-ran our main analysis but attempted to predict number of protective behaviors that improved (i.e. increased: sleep, healthy eating, physical activity [vs. decreased/remained same] and decreased: bingeing, alcohol use [vs. increased/remained same]). Outcome data were as follows: improvement in 0 behaviors (138, 19%), 1 behavior (202, 28%), 2 behaviors (180, 25%), 3 behaviors (120, 17%), 4 behaviors *69, 10%), 5 behaviors (14, 2%). In the first step of the model we only found evidence that age (*B* = -0.21, p < .001) and psychiatric illness (*B* = -0.22, p = .04) were significantly associated with number of behaviors improved, whereby older participants and participants with a psychiatric diagnosis were less likely to increase protective behaviors. In the second step of the model none of the psychological well-being measures were significant predictors and psychiatric diagnosis was no longer a significant predictor. Inclusion of psychological well-being measures did not significantly increase model fit (R squared change = .01, p > .05).

**Predicting change in individual protective behaviors since lockdown**

We used Multinomial Logistic Regressions to model changes in individual behaviors. We ran a model with demographic variables only (model 1) and then a model included depression, anxiety, stress and loneliness (model 2). Supplemental tables 2-6 report results from model 2.

Across individual behaviors results differed. As in the main analyses, having overweight or obesity was associated with some negative outcomes (e.g. decreased exercise, decreased healthy eating, increased bingeing on food), but there was also evidence that a sub-group of participants with overweight increased exercise. Consistent with main analyses, stress was associated with reduced sleep and increased bingeing. When loneliness was associated with outcomes, they tended to be positive (e.g. increased exercise) and this may reflect how people are coping with loneliness. Older people were less likely to show increased exercise, increased sleep and decreased alcohol use. See below for full detailed results.

*Exercise*

The demographic model unadjusted for psychological well-being variables was statistically significant (χ^2^(24) = 46.75, p = .004; AIC = 1491.69; BIC = 1610.86). The model adjusted for psychological well-being variables was statistically significant (χ^2^ (32) = 56.68, p = .005; AIC = 1507.45; BIC = 1663.29). There was limited evidence for overdispersion (Deviance χ^2^ (1412) = 1439.45). The individual predictors are shown in supplemental table 1. Having overweight (compared to normal weight) was associated with both decreased and increased exercise, depressive symptoms predicted a decline in exercise, whereas younger age and increased loneliness predicted increases in exercise.

*Supplementary Table 1: Multinomial Logistic Regression predicting reported decline or increase in exercise compared to no change.*

***Predictor*** ***Decline in Exercise Increase in Exercise***

*MOR LB95% UB95% MOR LB95% UB95%*

*Gender .860 .533 1.388 1.311 .822 2.090*

*Age .977 .954 1.001 .974 .952 .996**

*Ethnicity 1.468 .819 2.632 .964 .541 1.717*

*Education 1.496 .939 2.385 1.202 .770 1.876*

*Household Income 1.006 .996 1.016 1.007 .998 1.017*

*High risk condition 1.044 .606 1.800 .775 .454 1.324*

*Living alone 1.831 .917 3.656 1.678 .876 3.214*

*Psychiatric Condition .899 .548 1.477 .652 .403 1.055*

*COVID Diagnosis 1.270 .651 2.477 1.706 .902 3.228*

*Underweight 1.709 .501 5.827 2.090 1.172 3.726*

*Overweight 2.258 1.241 4.109** 2.090 1.172 3.726**

*Obese 1.187 .671 2.101 .981 .566 1.698*

*Loneliness .983 .963 1.004 .979 .959 .999**

*Depression 1.079 1.003 1.162* 1.049 .977 1.127*

*Anxiety .942 .857 1.036 .954 .870 1.046*

*Stress .990 .913 1.073 .988 .914 1.068*

*Legend: MOR = Multinomial Odds Ratio; LB95% = Lower Bound Confidence Interval; UB95% Upper Bound Confidence Interval; Gender (male reference); Ethnicity (white reference); Education (no degree reference); Household income (£1000s); High risk condition (low risk reference); Living alone (reference); Psychiatric Condition (absent reference); Living alone (alone reference); COVID diagnosis (absent reference). *p<05, p*<01*

*Sleep*

The demographic model unadjusted for psychological well-being variables was statistically significant (χ^2^(24) = 65.91, p < .001; AIC = 1484.42; BIC = 1603.60). The model adjusted for psychological well-being variables was statistically significant (χ^2^ (32) = 104.87, p < .001; AIC = 1472.56; BIC = 1628.39). There was limited evidence for overdispersion (Deviance χ^2^(1412) =1404.56). The individual predictors are shown in supplemental table 2. A COVID19 diagnosis was associated with both a decrease and increase in sleep, compared to no change. Greater stress was associated with reduced sleep, and there was a negative association between age and increased sleep compared to no change.

*Supplementary Table 2: Multinomial Logistic Regression predicting reported decline or increase in sleep compared to no change.*

***Predictor*** ***Decline in Sleep Increase in Sleep***

*MOR LB95% UB95% MOR LB95% UB95%*

*Gender .850 .525 1.374 1.045 .700 1.561*

*Age .990 .967 1.013 .959 .939 .978***

*Ethnicity .715 .380 1.345 1.343 .838 2.151*

*Education .836 .524 1.331 1.018 .684 1.516*

*Household Income 1.007 .998 1.016 .996 .989 1.004*

*High risk condition 1.491 .833 2.670 1.457 .890 2.387*

*Living alone .514 .249 1.064 .970 .517 1.819*

*Psychiatric Condition .899 .548 1.477 1.012 .654 1.565*

*COVID Diagnosis 2.281 1.124 4.627* 2.423 1.291 4.548***

*Underweight 1.368 .347 5.399 1.523 .478 4.851*

*Overweight 1.377 .802 2.365 1.176 .746 1.854*

*Obese 1.011 .565 1.809 .867 .526 1.429*

*Loneliness 1.018 .996 1.040 1.004 .987 1.023*

*Depression .984 .915 1.059 .999 .938 1.064*

*Anxiety 1.010 .916 1.114 1.016 .930 1.109*

*Stress 1.140 1.053 1.234** 1.027 .958 1.100*

*Legend: MOR = Multinomial Odds Ratio; LB95% = Lower Bound Confidence Interval; UB95% Upper Bound Confidence Interval; Gender (male reference); Ethnicity (white reference); Education (no degree reference); Household income (£1000s); High risk condition (low risk reference); Living alone (reference); Psychiatric Condition (absent reference); Living alone (alone reference); COVID diagnosis (absent reference).*

**p<05, p*<01*

*Healthy eating*

The demographic model unadjusted for psychological well-being variables was not statistically significant (χ^2^(24) = 33.73, p = .090; AIC = 1591; BIC = 1710). The model adjusted for psychological well-being variables was statistically significant (χ^2^ (32) = 56.77, p = .004; AIC = 1593; BIC = 1749). There was limited evidence for overdispersion (Deviance χ^2^ (1412) = 1525.70). The individual predictors are shown in supplemental table 3. Both overweight and obesity were associated with a decline in healthy eating.

*Supplementary Table 3: Multinomial Logistic Regression predicting reported decline or increase in healthy eating compared to no change.*

***Predictor*** ***Decline in Health Eat Increase in Health Eat***

*MOR LB95% UB95% MOR LB95% UB95%*

*Gender .866 .579 1.294 1.107 .738 1.659*

*Age .981 .961 1.001 .979 .959 1.000*

*Ethnicity .715 .380 1.345 1.184 .748 1.873*

*Education 1.020 .628 1.657 1.491 .998 2.229*

*Household Income 1.003 .995 1.011 1.002 .994 1.010*

*High risk condition 1.047 .651 1.683 .931 .571 1.519*

*Living alone .736 .396 1.366 .974 .516 1.835*

*Psychiatric Condition 1.070 .702 1.631 .808 .519 1.258*

*COVID Diagnosis .844 .497 1.431 1.005 .598 1.688*

*Underweight 1.006 .369 2.744 .922 .343 2.477*

*Overweight 1.623 1.023 2.574* 1.471 .934 2.316*

*Obese 2.016 1.232 3.300** 1.186 .698 2.017*

*Loneliness 1.012 .994 1.030 1.010 .992 1.028*

*Depression 1.009 .950 1.072 .947 .888 1.009*

*Anxiety .970 .895 1.051 1.056 .971 1.148*

*Stress 1.062 .995 1.134 1.003 .938 1.073*

*Legend: MOR = Multinomial Odds Ratio; LB95% = Lower Bound Confidence Interval; UB95% Upper Bound Confidence Interval; Gender (male reference); Ethnicity (white reference); Education (no degree reference); Household income (£1000s); High risk condition (low risk reference); Living alone (reference); Psychiatric Condition (absent reference); Living alone (alone reference); COVID diagnosis (absent reference).*

**p<05, p*<01*

*Binged on food*

The demographic model unadjusted for psychological well-being variables was statistically significant (χ^2^(24) = 39.92, p = .022; AIC = 1491; BIC = 1610). The model adjusted for psychological well-being variables was statistically significant (χ^2^ (32) = 80.71, p < .001; AIC = 1476; BIC = 1632). There was limited evidence for overdispersion (Deviance χ^2^ (1412) = 1408). The individual predictors are shown in supplemental table 4. Overweight (compared to normal weight) was associated with both decreased and increased binge eating compared to no change. Increased household income and loneliness were associated with decline in binge eating. Stress was associated with an increase in binge eating.

*Supplementary Table 4: Multinomial Logistic Regression predicting reported decline or increase in binge eating compared to no change.*

***Predictor*** ***Decline in Binge Eat Increase in Binge Eat***

*MOR LB95% UB95% MOR LB95% UB95%*

*Gender 1.230 .762 1.985 1.263 .861 1.852*

*Age .992 .968 1.016 .975 .956 .995**

*Ethnicity 1.423 .809 2.504 1.366 .862 2.163*

*Education .977 .605 1.579 .786 .537 1.151*

*Household Income 1.010 1.001 1.019* 1.007 .999 1.015*

*High risk condition .688 .374 1.263 1.004 .638 1.579*

*Living alone .734 .342 1.573 .634 .349 1.151*

*Psychiatric Condition 1.369 .814 2.303 1.055 .693 1.607*

*COVID Diagnosis 1.316 .706 2.452 1.021 .605 1.723*

*Underweight 1.698 .505 5.706 1.376 .489 3.873*

*Overweight 1.987 1.162 3.398* 1.634 1.046 2.554**

*Obese 1.326 .710 2.476 1.505 .928 2.440*

*Loneliness 1.026 1.005 1.049* 1.011 .994 1.029*

*Depression .927 .859 1.001 1.000 .942 1.061*

*Anxiety .983 .887 1.090 .975 .900 1.057*

*Stress 1.080 .995 1.172 1.118 1.047 1.194***

*Legend: MOR = Multinomial Odds Ratio; LB95% = Lower Bound Confidence Interval; UB95% Upper Bound Confidence Interval; Gender (male reference); Ethnicity (white reference); Education (no degree reference); Household income (£1000s); High risk condition (low risk reference); Living alone (reference); Psychiatric Condition (absent reference); Living alone (alone reference); COVID diagnosis (absent reference).*

**p<05, p*<01*

*Alcohol consumption*

The demographic model unadjusted for psychological well-being variables was statistically significant (χ^2^(24) = 110.89, p < .001; AIC = 1501; BIC = 1621). The model adjusted for psychological well-being variables was statistically significant (χ^2^ (32) = 124.06, p < .001; AIC = 1511; BIC = 1667). There was limited evidence for overdispersion (Deviance χ^2^ (1412) = 1443). The individual predictors are shown in supplemental table 5. Age was negatively associated with a decrease in alcohol use. Gender (male) was associated with increased alcohol use, as was COVID19 diagnosis, Ethnicity, presence of a high-risk condition, and household income.

*Supplementary Table 5: Multinomial Logistic Regression predicting reported decline or increase in binge eating compared to no change.*

***Predictor*** ***Decline in Alcohol con Increase in Alcohol con***

*MOR LB95% UB95% MOR LB95% UB95%*

*Gender 1.136 .762 1.693 1.826 1.183 2.819**

*Age .939 .917 .961** .988 .968 1.009*

*Ethnicity 1.080 .693 1.685 .444 .255 .775***

*Education .895 .602 1.329 .977 .648 1.472*

*Household Income 1.000 .992 1.008 1.012 1.004 1.020**

*High risk condition 1.241 .775 1.987 .545 .319 .932**

*Living alone 1.119 .583 2.147 .614 .329 1.148*

*Psychiatric Condition .665 .427 1.036 .785 .504 1.221*

*COVID Diagnosis 1.383 .801 2.390 2.259 1.310 3.896***

*Underweight 1.714 .672 4.376 .552 .136 2.241*

*Overweight 1.484 .932 2.362 1.483 .931 2.365*

*Obese .902 .535 1.522 1.099 .663 1.822*

*Loneliness 1.004 .986 1.022 .990 .972 1.009*

*Depression .955 .896 1.017 1.012 .950 1.077*

*Anxiety 1.063 .978 1.156 1.011 .930 1.100*

*Stress 1.026 .958 1.099 1.060 .991 1.134*

*Legend: MOR = Multinomial Odds Ratio; LB95% = Lower Bound Confidence Interval; UB95% Upper Bound Confidence Interval; Gender (male reference); Ethnicity (white reference); Education (no degree reference); Household income (£1000s); High risk condition (low risk reference); Living alone (reference); Psychiatric Condition (absent reference); Living alone (alone reference); COVID diagnosis (absent reference).*

**p<05, p*<01*
